# Supplementary material for: Mechanical disengagement of the cohesin ring
Source: Nat Struct Mol Biol. 2023 Oct 23;31(1):23–31. doi: 10.1038/s41594-023-01122-4 (PMC11377297; doi:10.1038/s41594-023-01122-4)
Supplement: Supplementary file 1 — Supplementary methods, methods references and video legends. [file 41594_2023_1122_MOESM1_ESM.pdf]

nature structural & molecular biology

Article https://doi.org/10.1038/s41594-023-01122-4

Mechanical disengagement of the cohesin ring

In the format provided by the authors and unedited

Supplementary Information

Mechanical disengagement of the cohesin ring

Martina Richeldi[1,2,3], Georgii Pobegalov[1,3], Torahiko L. Higashi[2,*], Karolina Gmurczyk[2], Frank Uhlmann[2]†, Maxim Molodtsov[1,3]†

[1]Biophysics and Mechanobiology Laboratory, and [2]Chromosome Segregation Laboratory, The Francis Crick Institute, London NW1 1AT, UK

[3]Department of Physics and Astronomy, University College London, London WC1E 6BT, UK

*Present address: Kamakura Research Laboratories, Chugai Pharmaceutical Co., Kamakura City, Kanagawa 247-8530, Japan

† Corresponding authors:

frank.uhlmann@crick.ac.uk (FU)

m.molodtsov@ucl.ac.uk (MM)

This file contains:

Supplementary Methods

Supplementary Methods References

Supplementary Video Legends

Supplementary methods

Construct generation

Wild type cohesin complexes

For generation of the cohesin *S. pombe* construct where force was applied *via* the head domain, SNAP-tag (for TMR attachment) and CLIP-tag (for biotin conjugation) sequences were fused to the Smc1^{Psm1} C-terminus and Smc3^{Psm3} C-terminus sequences, respectively, into previously constructed shuttle vectors YIplac211-Psm1/Psm3[1]. For generation of the cohesin *S. pombe* construct where force was applied *via* the hinge domain, SNAP-tag and CLIP-tag encoding sequences were fused to the hinge region of Smc1^{Psm1} (between R593 and G594, with one 8-residue linker flanking each side) and of Smc3^{Psm3} sequences (between D593 and A594, with one 8-residue linker flanking each side), respectively, in the same shuttle vector. The YIplac211-Psm1-SNAP/Psm3-CLIP and the YIplac128-Rad21/Psc3 vectors were sequentially integrated into a budding yeast plasmid at the *URA3* and *LEU2* loci, respectively.

Crosslinked cohesin complexes

For generation of the hinge-crosslinked cohesin *S. pombe* construct, the SpyTag-SpyCatcher system was used[2]. The *SpyCatcher* gene (138 residues) was introduced into the hinge region of Smc1^{Psm1} (between R593 and G594, with one 8-residue linker flanking each side), whereas the *SpyTag* gene (13 residues) was cloned into the hinge region of Smc3^{Psm3} (between D593 and A594, with one 8-residue linker flanking each side). Additional CLIP-tag and SNAP-tag sequences for biotin and TMR attachment, respectively, were introduced at the same positions as described for the cohesin variant where force was applied *via* the head domain and integrated into the same previously constructed shuttle vector. The YIplac211-Psm1-SNAP-SpyCatcher/Psm3-CLIP-SpyTag and the YIplac128-Rad21/Psc3 vectors were sequentially integrated into a budding yeast plasmid at the *URA3* and *LEU2* loci, respectively.

For generation of the Smc3^{Psm3}-kleisin crosslinked cohesin construct, the SpyTag-system was also used[2]. To generate a construct that would be directly comparable with the wild type complex, CLIP-tag and SNAP-tag sequences for biotin and TMR attachment, respectively, were introduced at the same positions as described for the cohesin variant where force was applied *via* the head domain. The *SpyTag* gene was inserted at two locations: i) at the N-terminus of the Scc1^{Rad21} subunit separated by a 24 amino-acid GS linker; and ii) at the Smc3^{Psm3} C-terminus after the CLIP-tag, separated by a 10 amino-acid linker. The sequences were integrated into the same previously constructed shuttle vector. The YIplac211-Psm1-SNAP/Psm3-CLIP-SpyTag and the YIplac128-Rad21-SpyTag/Psc3 sequences were then sequentially integrated into a budding yeast plasmid at the *URA3* and *LEU2* loci, respectively.

To generate the SpyCatcher-Linker-SpyCatcher crosslinker, the plasmid was constructed from two *SpyCatcher* gene sequences (addgene: 133447) separated by the unstructured amino acid sequence:

GSSGSGSLPGDSGTIIPAPGDSTTPLPGGVCISSPPSLPGGTAISPLSGDATIPPLPEGVGI
PSPSSLPGGTAIPLPGSARIPPPLPGSAGIPLPGEAGMPPLPGGPGIPFPGGPGIPGMGMP
FGSGSVPAAPVLPGTIIP.

This sequence contains 141 amino acid residues which separate the two SpyCatcher moieties. Assuming the average contour length of 1 amino acid residue in a disordered polypeptide to be ~ 0.4 nm, the expected length of this linker should be slightly over 50 nm.

We chose such a long linker because both SpyCatchers are expressed as a single polypeptide in the same direction, and we reasoned that a long linker length could facilitate the flexibility between the SpyCatchers and therefore improve chances of both proteins binding their

respective SpyTags regardless of their orientation, which should improve efficiency of the crosslinking.

This sequence was codon optimised for the expression in the *E. coli* BL21 strain and generated as a single geneblock (IDT DNA).

Cohesin complexes for visualisation of second DNA capture

For the second DNA capture visualisation experiments, a *S. pombe* cohesin construct lacking CLIP-tag for biotin attachment but bearing a SNAP-tag for fluorophore conjugation was generated. The absence of the biotin tag would ensure binding of the streptavidin-coated beads only to the second DNA substrate, whereas a dye alternative to TMR (excited by a 531 nm wavelength laser) was selected to guarantee that the fluorescent signals of the second DNA plasmid (excited by a 488 nm wavelength laser) and that of cohesin would not bleed into one another's visualisation channels. The SNAP-tag sequence for conjugation of the LD655 dye (excited by a 647 nm wavelength laser) was fused to the $Scc1^{Rad21}$ C-terminus.

<u>Protein expression, purification and labelling</u>

Fission yeast cohesin tetramer complexes, $Scc2^{Mis4}$-$Scc4^{Ssl3}$, $Pds5^{Pds5}$ and $Wpl1^{Wapl}$ were expressed and purified as described in previously published protocols[1].

Briefly, fission yeast cohesin tetramer complexes were expressed in W303 background budding yeast strains. Cultures were grown at 30 °C in YP medium (2% peptone and 1% yeast extract) with the addition of 2% raffinose until the optical density at 595 nm reached 1.0-1.5 a. u., at which point protein expression was induced by addition of 2% galactose for 4h. After centrifugation, cells were re-suspended in an equal volume of CLH buffer (50 mM HEPES/KOH, Ph 7.5, 1 mM DTT, 300 mM NaCl, 20% (v/v) glycerol, 0.5 mM PMSF and

protease inhibitor cocktail, frozen using liquid nitrogen and disrupted in a freezer mill. The cell powder was thawed on ice, then 2x the volume of CLH buffer was added for resuspension. The lysate was clarified at 4 °C by centrifugation at 45,000g for 30 minutes and then at 200,000g for 60 minutes. The clarified lysate was mixed with IgG sepharose (GE Healthcare) and 10 µg/ml of RNase A at 4 °C for 3 h. The resin was washed with 15 bed volumes of H buffer (25 mM HEPES/KOH, pH 7.5, 0.5 mM TCEP, 10% (v/v) glycerol) containing 300 mM NaCl, and then with 15 bed volumes of H buffer without PMSF. The resin was then suspended in two bed volumes of L buffer (20 mM Tris/HCl, pH 7.5, 0.5 mM tris(2-carboxyethyl)phosphine (TCEP), 10% (v/v) glycerol) containing 250 mM NaCl, 10 µg/ml RNase A and 5 units/ml of PreScission protease (GE Healthcare) and incubated overnight at 4 °C. For elution, two volumes of L buffer were added to the eluate before loading onto a HiTrap Heparin HP column (GE Healthcare). Bound proteins were eluted with steps of 300 mM, 600 mM and 1 M NaCl in L buffer. Cohesin was retrieved in the 600 mM NaCl fraction. This fraction was pooled and applied to a Superose 6 10/300 GL gel filtration column (GE Healthcare) that was equilibrated in L buffer containing 200 mM NaCl. The peak fractions were concentrated to 500 µl final volume by ultrafiltration (100 kDa, Amicon Ultra, Millipore).

For SNAP-tag labelling, cohesin was incubated with 4 µM of the fluorescent dye conjugated with benzylguanine, 1 mM DTT (Sigma-Aldrich) and 10% (v/v) Tween20 at 25 °C for 1 h. The fluorescent dyes used in this study were either TMR (New England BioLabs) or LD655 (Lumidyne Technologies). For CLIP-tag labelling, cohesin was supplemented with 8 µM BC-biotin (New England BioLabs) at 4 °C for 16 h. The labelled cohesin was then applied to a Superose 6 10/300 GL (GE Healthcare) gel filtration column. Peak fractions were pooled and concentrated by ultrafiltration for bulk assay but were not concentrated for single-molecule experiments. The efficiency of fluorescent labelling was determined by recording an

absorbance spectrum between 220-800 nm in 1 nm increments using a V-550 Spectrophotometer (Jasco). The samples were then aliquoted, frozen in liquid nitrogen and stored at -80°C.

The His-TEV-SpyCatcher-Linker-SpyCatcher crosslinker (used with $Smc3^{Psm3}$-kleisin cohesin) was expressed in *E. coli* pLys BL21 cells overnight at 18°C in LB medium containing 0.5 mM IPTG and 0.1 mg/ml ampicillin. Cells were collected and lysed in lysis buffer (50 mM HEPES pH 7.5, 400 mM NaCl, 20 mM Imidazole plus complete protease inhibitor cocktail (Roche)). The clarified lysate was incubated with NiNTA beads (Qiagen) for 90 minutes and eluted with elution buffer (20 mM HEPES, 100 mM NaCl, 400 mM Imidazole, pH 7.5). The sample was desalted into 50 mM HEPES pH 7.5, 150 mM NaCl and incubated with TEV protease overnight. Next, TEV protease and the cleaved off His-tag were removed by incubation with NiNTA beads. The supernatant was aliquoted, frozen in liquid nitrogen and stored at -80°C.

Western blotting

Fission yeast cohesin was detected using mouse monoclonal anti-HA antibody (Sigma-Aldrich) and anti-mouse IgG (HRP-conjugated) antibody (GE Healthcare). Both antibodies were used in a 1:10,000 dilution in PBS, in a final volume of 20 ml. Chemiluminescence was detected by using ECL Prime Western blotting detection reagents (GE Healthcare) and signals quantified using a ChemiDoc MP imaging system (BioRad).

Supplementary Methods References

1 Murayama, Y. & Uhlmann, F. Biochemical reconstitution of topological DNA binding by the cohesin ring. *Nature* **505**, 367-371 (2014).

2 Keeble, A. H. *et al.* Evolving Accelerated Amidation by SpyTag/SpyCatcher to Analyze Membrane Dynamics. *Angew Chem Int Ed Engl* **56**, 16521-16525 (2017).

Supplementary Video Legends

Supplementary Video 1. Cohesin slides of enzymatically cleaved surface tethered λ-DNA. Upon cleavage of the tethered λ-DNA (stained with Sytox Orange) by single-site restriction enzyme XhoI, the fluorescently-labelled cohesin slides off the DNA.

Supplementary Video 2. Force application to cohesin on DNA in x and y directions. This video shows force applied to a single cohesin on λ-DNA either along or perpendicularly to the DNA. Relates to Fig. 2a.

Supplementary Video 3. Force application to a single cohesin on DNA. Time lapse video shows force application to a single cohesin on λ-DNA via optically trapped bead until it ruptures. Relates to Fig. 2b.

Supplementary Video 4. Spontaneous dissociation of the second ssDNA from cohesin. This time lapse video shows transient second ssDNA capture and release. Relates to Extended Data Fig. 7c.

Supplementary Video 5. Force application to the ssDNA-cohesin-DNA interaction. This time lapse video shows force application to the bead attached to the second ssDNA substrate. ssDNA is bound to λ-DNA via a single cohesin complex. The bead detachment from the λ-DNA corresponds to the rupture of the cohesin ring. Relates to Extended Data Fig. 8a.

Supplementary Video 6. Force application to the dsDNA-cohesin-DNA interaction. This time lapse video shows force application to the bead attached to the second dsDNA substrate. dsDNA is bound to λ-DNA by a single cohesin complex. The bead detachment from the λ-DNA corresponds to the rupture of the cohesin ring. Relates to Fig. 5b.
