# Supplementary material for: Mechanical disengagement of the cohesin ring
Source: Nat Struct Mol Biol. 2023 Oct 23;31(1):23–31. doi: 10.1038/s41594-023-01122-4 (PMC11377297; doi:10.1038/s41594-023-01122-4)
Supplement: Supplementary file 2 — Reporting Summary [file 41594_2023_1122_MOESM2_ESM.pdf]

nature portfolio

Corresponding author(s): Maxim Molodtsov

Last updated by author(s): Aug 25, 2023

Reporting Summary

Nature Portfolio wishes to improve the reproducibility of the work that we publish. This form provides structure for consistency and transparency in reporting. For further information on Nature Portfolio policies, see our Editorial Policies and the Editorial Policy Checklist.

Statistics

For all statistical analyses, confirm that the following items are present in the figure legend, table legend, main text, or Methods section.

n/a	Confirmed	
☐	☒	The exact sample size (n) for each experimental group/condition, given as a discrete number and unit of measurement
☐	☒	A statement on whether measurements were taken from distinct samples or whether the same sample was measured repeatedly
☐	☒	The statistical test(s) used AND whether they are one- or two-sided<br>*Only common tests should be described solely by name; describe more complex techniques in the Methods section.*
☐	☒	A description of all covariates tested
☐	☒	A description of any assumptions or corrections, such as tests of normality and adjustment for multiple comparisons
☐	☒	A full description of the statistical parameters including central tendency (e.g. means) or other basic estimates (e.g. regression coefficient) AND variation (e.g. standard deviation) or associated estimates of uncertainty (e.g. confidence intervals)
☐	☒	For null hypothesis testing, the test statistic (e.g. F, t, r) with confidence intervals, effect sizes, degrees of freedom and P value noted<br>*Give P values as exact values whenever suitable.*
☐	☒	For Bayesian analysis, information on the choice of priors and Markov chain Monte Carlo settings
☒	☐	For hierarchical and complex designs, identification of the appropriate level for tests and full reporting of outcomes
☒	☐	Estimates of effect sizes (e.g. Cohen's d, Pearson's r), indicating how they were calculated

Our web collection on statistics for biologists contains articles on many of the points above.

Software and code

Policy information about availability of computer code

Data collection	The JPK optical trap control software (v 6.1) was employed to operate the optical trap and collect force-distance data. The Andor Solis (v 4.31) software was used to acquire and record microscopy data simultaneous with force application/measurements by the JPK software. The Nikon NIS-Elements (v 5.41) software was used to image microscopy data obtained without force application experiments.
Data analysis	The JPK Processing (v 6.1) Software was used to visualise force-distance curves and extract the magnitudes of rupture forces. FIJI (ImageJ, v 1.54) was used for visualisation and image analysis. Custom-made MATLAB codes were used to for single-molecule fluorescence intensity analysis, force rupture determination and visualisation, histogram fitting, statistical testing and simulations. All codes are publicly available on GitHub (https://github.com/FrancisCrickInstitute/DNA_Cohesin_MMC).

For manuscripts utilizing custom algorithms or software that are central to the research but not yet described in published literature, software must be made available to editors and reviewers. We strongly encourage code deposition in a community repository (e.g. GitHub). See the Nature Portfolio guidelines for submitting code & software for further information.

Data

Policy information about availability of data

All manuscripts must include a data availability statement. This statement should provide the following information, where applicable:

- Accession codes, unique identifiers, or web links for publicly available datasets
- A description of any restrictions on data availability
- For clinical datasets or third party data, please ensure that the statement adheres to our policy

Source data used for the generation of main figures is provided with this paper. Example data is included with the software codes provided freely on GitHub. The rest of the raw data will be made available by the authors upon request.

Research involving human participants, their data, or biological material

Policy information about studies with human participants or human data. See also policy information about sex, gender (identity/presentation), and sexual orientation and race, ethnicity and racism.

Reporting on sex and gender	N/A
Reporting on race, ethnicity, or other socially relevant groupings	N/A
Population characteristics	N/A
Recruitment	N/A
Ethics oversight	N/A

Note that full information on the approval of the study protocol must also be provided in the manuscript.

Field-specific reporting

Please select the one below that is the best fit for your research. If you are not sure, read the appropriate sections before making your selection.

☒ Life sciences ☐ Behavioural & social sciences ☐ Ecological, evolutionary & environmental sciences

For a reference copy of the document with all sections, see nature.com/documents/nr-reporting-summary-flat.pdf

Life sciences study design

All studies must disclose on these points even when the disclosure is negative.

Sample size	Sample size was not predetermined. We collected data until further increase in the sample size either did not directly improve variances of the k0 and delta parameters inferred from the data or until the bootstrap analysis indicated that the further increase was not expected to change the significance of the difference between parameters inferred from experiments in different conditions.
Data exclusions	Force-distance data exhibiting more than one peak were excluded from total rupture forces distributions. The rationale behind the exclusion of these data is that multiple force-distance peaks likely indicate more than one cohesin molecule connecting the beads and DNA. As their single-nature could not be confirmed, accurate analysis was not possible.
Replication	The number of repeat measurements made is stated in the figure legends. All experiments were repeated at least three times (up to one hundred times), performed on different days and in independent flow cells.
Randomization	Single-molecule data with the same conditions for DNA substrates were inherently randomized and no other special randomization was implemented.
Blinding	Investigators were not blinded. All samples were prepared on the same day the measurement was performed and prepared by the same author collecting the data.

Reporting for specific materials, systems and methods

We require information from authors about some types of materials, experimental systems and methods used in many studies. Here, indicate whether each material, system or method listed is relevant to your study. If you are not sure if a list item applies to your research, read the appropriate section before selecting a response.

Materials & experimental systems

n/a	Involved in the study
☐	☒ Antibodies
☒	☐ Eukaryotic cell lines
☒	☐ Palaeontology and archaeology
☒	☐ Animals and other organisms
☒	☐ Clinical data
☒	☐ Dual use research of concern
☒	☐ Plants

Methods

n/a	Involved in the study
☒	☐ ChIP-seq
☒	☐ Flow cytometry
☒	☐ MRI-based neuroimaging

Antibodies

Antibodies used

For single-molecule experiments:
Antibody: Anti-Digoxigenin-AP, Fab fragments
Supplier: Roche
Catalogue number: 11093274910

For Western blotting:
Antibody: Mouse monoclonal anti-V5 tag (anti-Pk tag)
Supplier: Bio-Rad
Catalogue Number: MCA1360

Antibody: Mouse monoclonal anti-HA tag
Supplier: Sigma-Aldrich
Catalogue Number: 11583816001

Antibody: Anti-mouse IgG (HRP conjugated)
Supplier: GE Healthcare
Catalogue Number: NA931

Antibody dilutions used for Western blotting were 1:10000. Other dilutions are indicated in the Methods and Supplementary Methods of the Paper.

Validation

For the anti-Digoxigenin-AP, see supplier's website, containing relevant references of validation: https://www.sigmaaldrich.com/GB/en/product/roche/11093274910

For the mouse monoclonal anti-V5 antibody, see supplier's website, under "product specific references: https://www.bio-rad-antibodies.com/monoclonal/viral-v5-tag-antibody-sv5-pk1-mca1360.html?f=purified&JSESSIONID_STERLING=5864A39766958B0AB4D28A9E7712D453.ecommerce1&evCntryLang=UK-en&EU_COOKIE_PREFS=111&cntry=UK&thirdPartyCookieEnabled=true

For the mouse monoclonal anti-HA antibody, see supplier's website, containing relevant references of validation: https://www.sigmaaldrich.com/GB/en/product/roche/roaha
