# Supplementary material for: Mechanical disengagement of the cohesin ring
Source: Nat Struct Mol Biol. 2023 Oct 23;31(1):23–31. doi: 10.1038/s41594-023-01122-4 (PMC11377297; doi:10.1038/s41594-023-01122-4)
Supplement: Supplementary file 16 — Unprocessed gels. [file 41594_2023_1122_MOESM16_ESM.pdf]

b

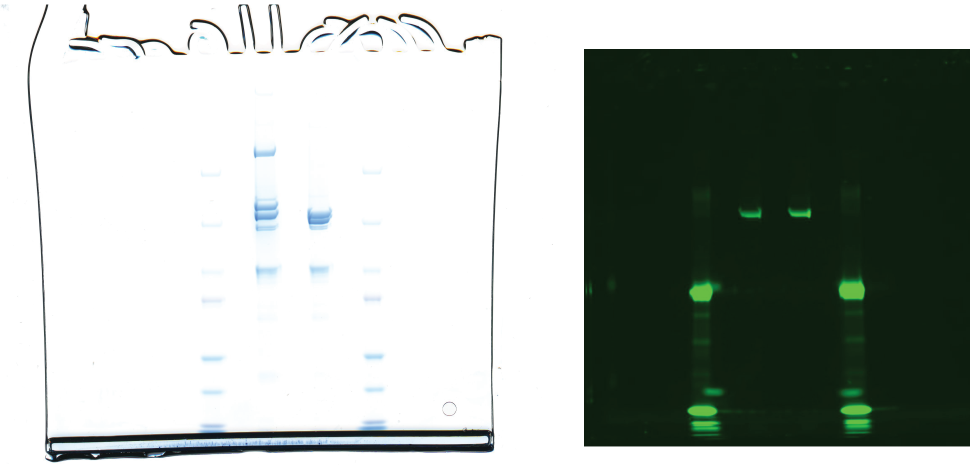

d

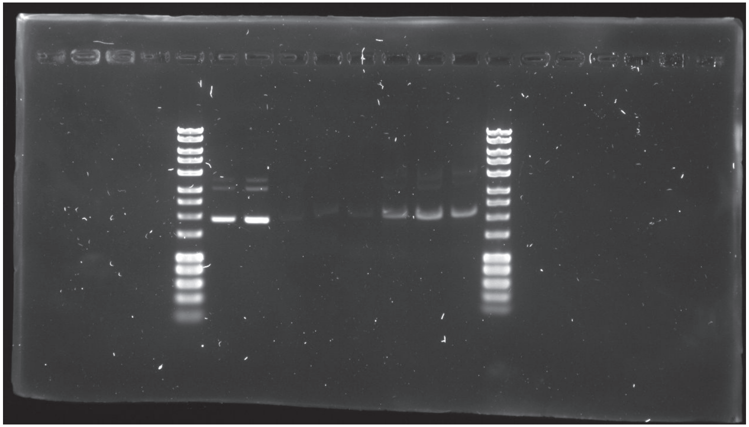

f

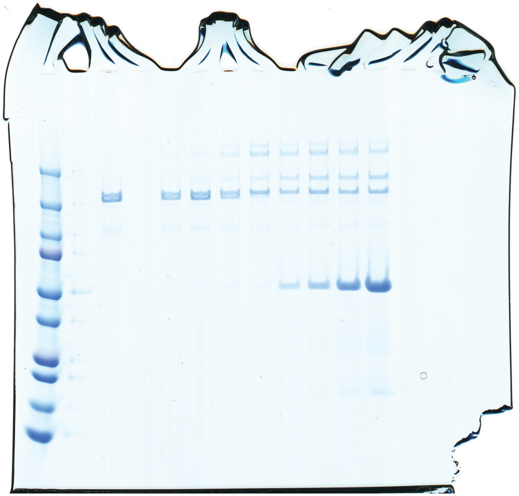

Extended Data Figure 1. Characterisation of the recombinant cohesin complexes and topological DNA loading
