# Supplementary figures and images for: Mechanical disengagement of the cohesin ring
Source: Nat Struct Mol Biol. 2023 Oct 23;31(1):23–31. doi: 10.1038/s41594-023-01122-4 (PMC11377297; doi:10.1038/s41594-023-01122-4)

**b**

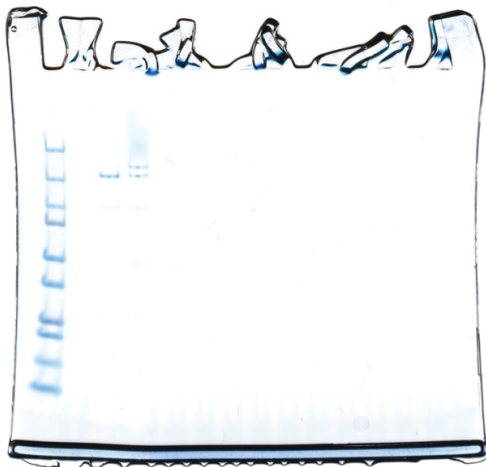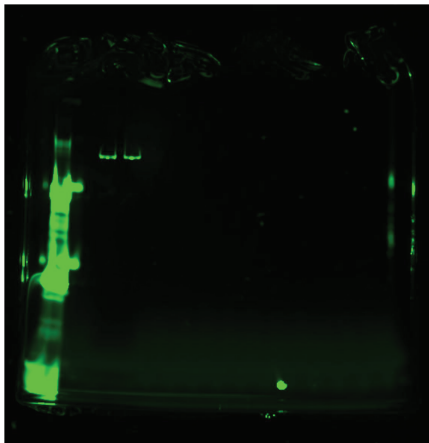

Extended Data Figure 3. Characterisation of cohesin with a biotin handle at the hinge

Supplement: Supplementary file 18 — Unprocessed gels. [file 41594_2023_1122_MOESM18_ESM.pdf]

**b**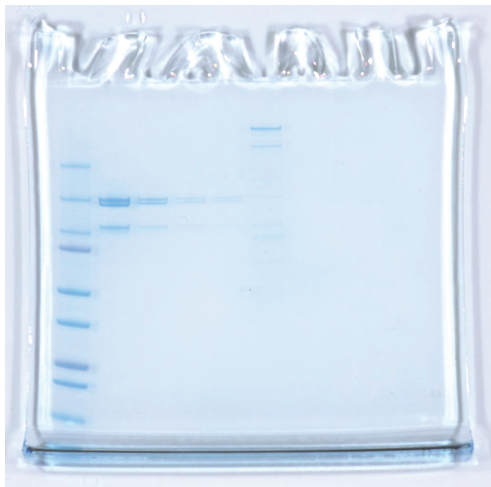**c**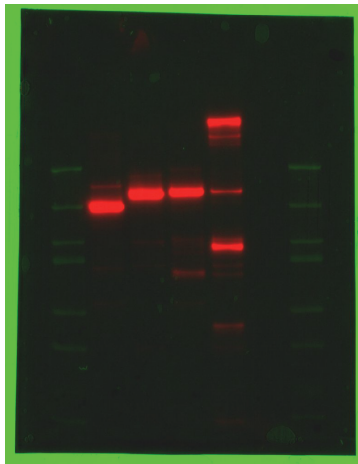

Extended Data Figure 5. Characterisation of the hinge-crosslinked cohesin complex.

Supplement: Supplementary file 20 — Unprocessed gels. [file 41594_2023_1122_MOESM20_ESM.pdf]
